# Enabling Large Model Inference in Dynamic and Heterogeneous Mobile Robots by Incorporating Collaborative Inference and Model Context Protocol

**DOI:** 10.3390/s26216976

**Published:** 2026-11-04

**Authors:** Qiang Han, Josh Dafoe, Bo Chen

**Affiliations:** Department of Computer Science, Michigan Technological University, Houghton, MI 49931, USA

**Keywords:** autonomous mobile robots, large language model, collaborative inference, model context protocol, dynamics, heterogeneity

## Abstract

Modern robotic systems increasingly rely on transformer-based AI models to process sensor input and support high-level decision making. However, due to limited GPU resources in each ego robot, it is difficult to run a large model locally. Outsourcing the model inference to cloud providers could address this issue, which however suffers from limitations including unreliable network connection, high latency, and high monetary cost. In this work, we have developed an effective decision making framework for robotic systems by leveraging a few state-of-the-art technologies, such as collaborative inference and model context protocol (MCP). Our framework seamlessly integrates collaborative inference between edge servers and mobile robots, supporting inference of large models by relying on local resources instead of remote cloud-based resources. It excels in a few feature designs. First, it is designed for mobile robot deployments where the available computing resources may change over time. This is achieved by introducing “anchors”, adding redundant nodes to hold the model partitions, and switching among model variants of different sizes. Second, it can allow heterogeneous robotic peers to seamlessly join the system through MCP, a standardized interface for modern AI applications. We have implemented a prototype of the proposed framework and experimentally validated its efficiency.

## 1. Introduction

In recent years, the autonomous robotics software stack has been shifting toward more integrated designs. In particular, many approaches have transitioned from traditional modular pipelines—comprising separate sensing, perception/modeling, prediction, planning, and control components [1,2]—to end-to-end (e2e) frameworks. This approach uses a monolithic differentiable model that takes sensor data as input and directly outputs robot actions or control signals. This end-to-end approach reduces complexity while addressing optimization problems and cross-component information loss [3,4,5]. However, a remaining fundamental challenge is determining which model(s) to place within the end-to-end architecture.

An emerging approach is dynamic model selection, where the model that provides robot control is selected based on the specific task. In general, there are two approaches. One is to integrate the selection of sub-models within a mixture-of-experts e2e model [6,7], and the other approach maintains multiple separate e2e models and uses an external selector to dynamically activate the appropriate model(s) based on the current context or state [8,9,10,11].

Large language models have recently been applied to robotic systems [12,13]. Due to the significant general knowledge base of these models, they have high zero-shot learning capabilities, and their textual understanding enables explainability and adaptability to user instructions. Scaling laws [14] indicate that models of larger size will generally unlock improved capabilities, which, however, require significantly more GPU memory resources. For example, the PaLM-E embodied multimodal model has a version of up to 562 billion parameters [13] and hosting the entire model in FP16 requires more than 1 TB of GPU memory.

Because local computing resources of robotic systems are limited, the available choices are usually restricted to smaller, less capable models. Cloud computing can be used to enable larger models to be used in robotic systems [15,16]. However, cloud-based inference may suffer from high network latency [17,18]. In addition, since maintaining a dedicated large-scale computing infrastructure is costly, relying on cloud computing typically incurs high usage fees.

To support the execution of larger models in the ego robot while eliminating its reliance on the cloud resources, we propose a distributed architecture that utilizes the compute and memory resources of *nearby* robots and edge servers. In contrast to cloud-based infrastructure, nearby robots and edge servers are much closer to the ego robot, thereby providing an opportunity for significant latency reduction. Most importantly, this can reduce monetary costs by fully utilizing available local resources, which are typically cheaper compared to the expensive dedicated cloud infrastructures.

To enable model inference using the computing resources pooled locally, we utilize a collaborative inference design, which forms a “virtual inference server” by (1) dividing a single AI model into multiple partitions and (2) distributing the model partitions across multiple inference servers. In this architecture, GPU-equipped edge servers or robots can serve as *inference servers*, providing services in exchange for potential rewards (where a reward can serve as an asset in the network) from the network. However, robots are heterogeneous (e.g., we consider a diverse set of autonomous mobile robots such as humanoids, robot dogs, and robot cars which are available in the wild) and dynamic (the robots are moving all the time), creating a few new challenges when integrating collaborative inference for high-level decision making:

First, we rely on locally available resources (e.g., the GPU resources available in mobile robots) which may change over time, potentially interrupting the ongoing collaborative inference services; additionally, it is non-trivial to coordinate the robots which are equipped with diverse hardware and software components in the collaborative inference process. To enable collaborative inference with dynamic nodes, we use the edge server as the orchestrator for each collaborative inference task. In this way, both the inference servers and *inference clients* (i.e., robots that use the inference services) have stable anchor points in the network. In addition, to allow heterogeneous robots to participate transparently in the collaborative inference process, we adapt the model context protocol (MCP) [19], an open protocol that was originally designed to support seamless integration between LLM applications and external data sources/tools. Specifically, our orchestrator server implements a few unique MCP “tools” so that the inference servers and clients can interact with these tools via the standardized MCP protocol, regardless of their diverse hardware and software configurations. This MCP-based standard interface offers significant advantages in real-world deployments of heterogeneous devices: inference servers can easily register with or unregister from the orchestrator servers, and inference clients can easily access the available inference services through the orchestrator servers, even without prior knowledge of each other. To provide the best services, and hence optimize potential rewards, the inference servers should dynamically register with the nearest orchestrators, which helps minimize the inference latency.

Second, the collaborative inference relies on the model partitions distributed to different peers, which, however, may leave in our setting. To accommodate these dynamics, we can re-distribute the model partitions once the topology is changed, but this immediate solution is highly inefficient. To address this issue, for a given AI model, we maintain a few versions different in model size, and the model version being used at the time is determined by the resources available during a certain period. Specifically, if there are more robots (determined by a threshold) nearby, we will use a larger model and distribute the model partitions to mobile robots; otherwise, we will switch to a smaller model. For the larger model, as mobile robots may unexpectedly leave the current orchestration region while actively hosting model partitions, immediately redistributing the model after every such departure can introduce a substantial delay. We therefore introduce a predictive backup mechanism, in which the orchestration server monitors the active inference robots and actively replicates model partitions hosted in those robots that are most likely to leave soon. When they actually leave, their partitions can be immediately served by the backup robots without interrupting the ongoing collaborative inference process. For the smaller models, they are mostly distributed to a small number of edge servers that are not sensitive to node dynamics.

**Contributions**. Our major contributions are summarized below:

- We have proposed an effective decision-making framework for robotic systems, integrating collaborative inference and the model context protocol to support large model inference using solely the local resources, eliminating the reliance on the remote cloud resources.
- Our framework is particularly designed for autonomous mobile robots. We evaluate collaborative inference under controlled environments.
- We have implemented a prototype of the proposed framework and have experimentally demonstrated its efficiency.

## 2. Background

### 2.1. Autonomous Mobile Robots

Autonomous mobile robots are mobile robotic systems that can perceive their environment, make real-time decisions, and perform actions without continuous human control. Popular autonomous mobile robots include humanoid robots, mobile ground robots, drones, autonomous vehicles, etc. Recent studies have integrated large language models into robotic systems for tasks such as perception, planning, and action generation [13,20,21,22]. Larger models can help mobile robots interpret complex environments and perform diverse tasks, but such models also require substantial computing and memory resources. However, the computing resources available on a mobile robot are limited by its hardware capacity. As a result, an autonomous mobile robot may not have sufficient GPU memory to execute a large model locally. Mobile robots may also join, leave, or move between network regions during operation. Consequently, both the available computing resources and the network topology can change over time. To utilize these dynamic resources, our framework allows a mobile robot to request model inference as an inference client or contribute its computing resources as an inference server.

### 2.2. Collaborative Inference

Deep neural network based AI systems usually rely heavily on GPUs to efficiently perform operations on large matrices in parallel. In particular, large transformer based models such as large language models require significant amounts of GPU RAM to hold tens or even hundreds of billions of parameters during execution. Therefore, limited local computing resources are often not sufficient to hold the entire LLM model in GPU RAM at one time. An option is to use regular RAM and storage as a cache for the GPU [23], but this can cause significant inference delay. Recent work has introduced collaborative inference [24], in which the entire LLM model is partitioned into transformer blocks, and sets of contiguous blocks are hosted on different servers. To perform an inference pass, activations are forwarded through the blocks in order, with forwarding between servers when needed.

### 2.3. Model Context Protocol (MCP)

In distributed AI systems, applications often interact with heterogeneous inference services deployed across different hardware and administrative domains. Without a standardized interface, clients must customize the integration logic for different service implementations. The model context protocol (MCP) is an interface specification designed to standardize interactions between AI applications and external tools or data sources [19]. The model context protocol follows a client-server architecture where the server provides context and the client maintains a connection to the server to obtain context. It adopts a JSON-RPC based messaging format and introduces a tool-oriented abstraction, where services are exposed as callable capabilities. Through this abstraction, clients can discover the available tools, resources and prompts, and invoke them using a consistent interface, without requiring knowledge of the underlying implementation details. By decoupling application logic from service-specific communication mechanisms, MCP provides a unified interaction layer that can be applied in heterogeneous and distributed computing environments.

The MCP protocol mainly defines three types of primitives that an MCP server can expose:

- *Tools*: These primitives are used to expose executable functions (e.g., create a file, send a message, query a database, etc.) that can be invoked by AI applications.
- *Resources*: These primitives are typically used to expose data sources, e.g., database records, docs, files, logs, etc.
- *Prompts*: These primitives are used to expose reusable and parameterized prompt templates (e.g., few-shot examples, review prompts, summarization prompts, etc.) that can help structure the interaction with LLMs.

This work mainly implements MCP “tools” to handle various requests, such as registration, deregistration, and inference requests, issued by autonomous mobile robots [25].

## 3. Design

In our design, we consider a distributed network consisting of semi- or fully-autonomous robots and edge servers equipped with GPUs. The autonomous control software stack utilizes an LLM-based approach with multiple model candidates. Additionally, the software stack includes a runtime model-selection module that chooses which model to use for robot control based on the operating context, network conditions, and currently available computing resources. Unfortunately, integrating large language models (LLMs) into the robot’s computing systems is infeasible locally. Thus, in order to integrate LLMs into the robot control systems, we propose collaboration between nearby robots and edge servers to create a “virtual” inference server that exposes its available models as candidates.

**Figure 1:**
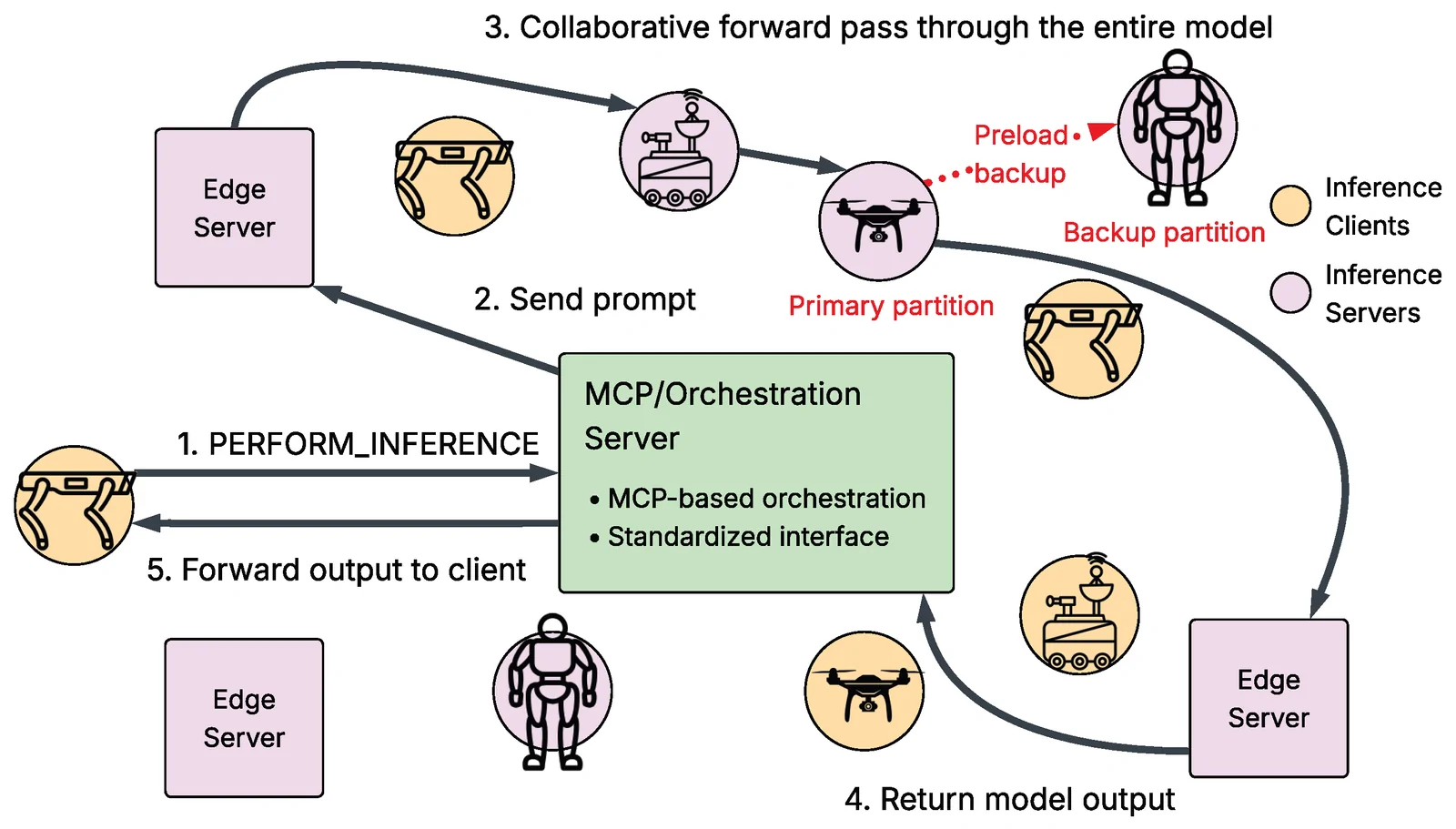
An overview of our system architecture and illustration of an inference pass.

Figure 1 presents the overall system architecture and illustrates the execution of an inference request. A robot client sends a request to a nearby MCP orchestration server. The orchestration server selects an available model and coordinates the execution of its partitions on multiple inference servers. Intermediate activations are forwarded between servers according to the selected inference path, and the final response is returned to the robot client. The orchestration server also maintains the current network state.

The design addresses a few critical obstacles. First, dynamic topology arises because mobile robots may join, leave, or move between network regions, causing the set of available and optimal inference servers to change over time. We overcome this obstacle through MCP-based orchestration, which provides stable access points and standardized interfaces for client discovery, server registration, and inference requests. The design details can be found in Section 3.1. Second, dynamic resource availability arises because the aggregated GPU memory, compute capacity, model availability, and network latency of the system vary over time. We overcome this obstacle through dynamic model selection and redistribution, allowing the orchestration server to determine when model reconfiguration should occur and which models should be deployed. The design details will be presented in Section 3.2. Third, an active mobile robot may leave the current orchestration region while hosting model partitions, which can interrupt the current inference process. We address this issue by introducing a predictive backup mechanism, which proactively replicates partitions hosted by robots that are more likely to leave. The design details are presented in Section 3.3.

### 3.1. Orchestration Server and MCP Interface

To address the first challenge, we introduce orchestration servers that provide a standard interface for both clients and inference servers to interact with the network, using the MCP protocol. By establishing a set of servers with fixed locations in the network, this strategy allows clients to offload the complexity of peer discovery and selection. In addition, inference servers then have a standard interface to register with the network and advertise their available resources, enabling the MCP servers to have a unified view of available network resources.

We adopt MCP primarily to provide a standardized tool interface for LLM applications. In the proposed framework, device-specific and backend-specific interfaces can be wrapped as MCP tools, allowing clients to access inference and orchestration capabilities through a common interface. Petals remains responsible for distributed model execution. The registration and heartbeat operations described below are application-specific tools defined by our framework, rather than built-in MCP operations.

The MCP server presents the following interface to the inference servers:

- REGISTER_SERVER: This request is used to register the caller as a collaborative inference server. During registration, the caller should indicate the maximum GPU memory they can dedicate to the collaborative inference service, the floating point operations per second, and the set of downloaded models.
- DEREGISTER_SERVER: If an inference server intentionally deregisters, it should call this procedure, in order to immediately update the MCP server’s network view.
- SERVER_HEARTBEAT: This procedure should be periodically called by inference servers, so that the MCP server can maintain an up-to-date network view. If an inference server fails to generate a heartbeat within some time threshold *T*, then the MCP server will automatically de-register the server. Importantly, information may be passed between the MCP server and inference servers during the heartbeat, if needed. For example, the MCP server may provide a list of registered inference servers and the inference servers may report the latency between other inference servers.

The MCP server presents the following interface to robot clients:

- QUERY_MODELS: Using this request, the client may query the MCP server to retrieve the set of models available for collaborative inference.
- PERFORM_INFERENCE: This is the procedure for calling into the remote inference service. The client should provide a requested model, prompt, and client ID. The MCP server will schedule the inference pass among the available resources, acting as a collaborative inference client. Figure 1 illustrates the steps during an inference pass.
- QUERY_RTT: This queries the round trip time between the robot client and MCP server. When receiving this command, the MCP server simply returns the request so that the client can measure the RTT.

When choosing an appropriate MCP server, the client will call QUERY_RTT on several nearby servers, and choose the one with the lowest latency. Note that the MCP servers themselves may optimize the inference path based on latency values received when calling SERVER_HEARTBEAT, using techniques similar to those introduced by Mohammed et al. [26]. However, such resource redistribution among the inference servers should be performed sparingly, because swapping new model blocks into GPU RAM is very time consuming.

**Figure 2:**
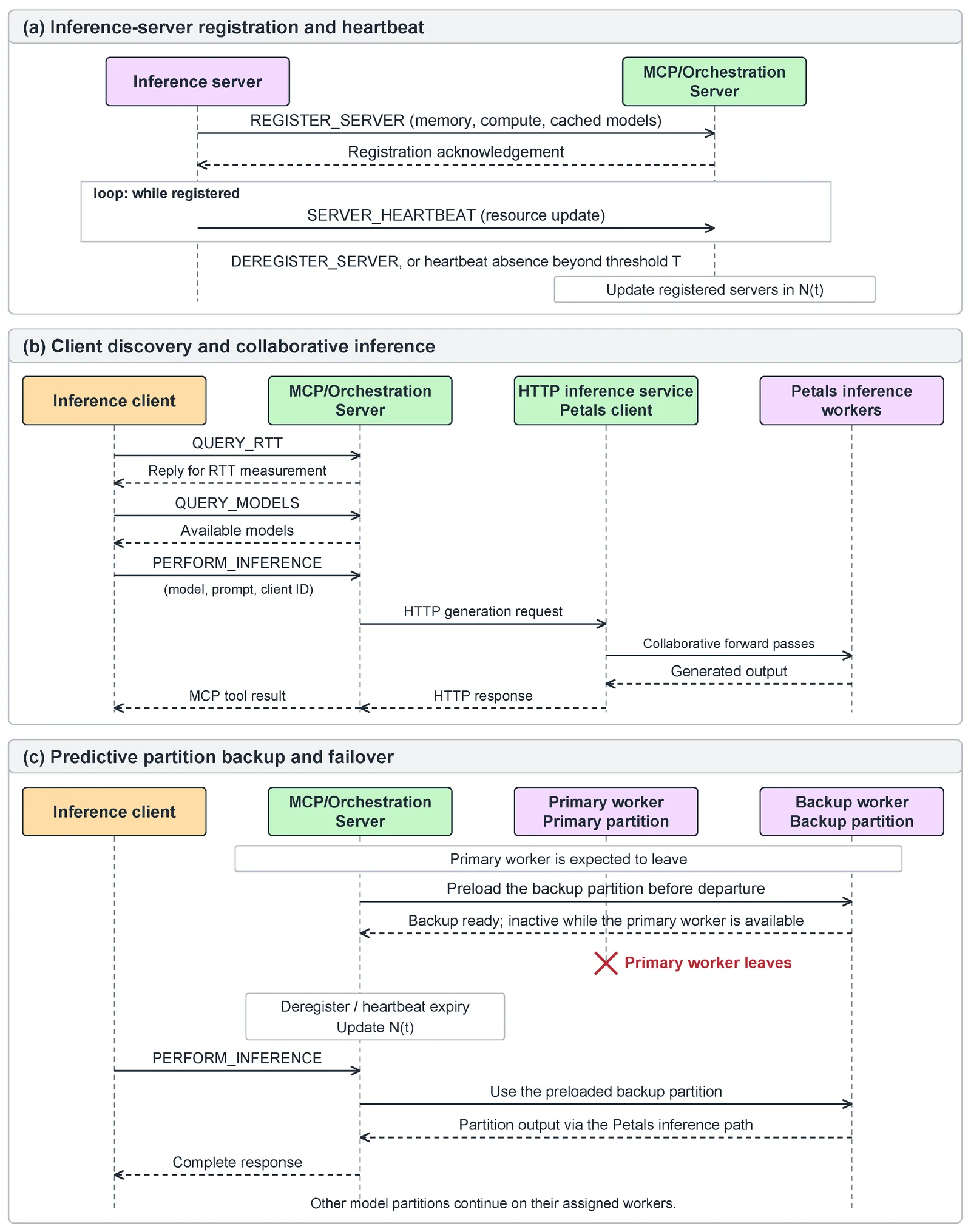
Sequence diagram of MCP orchestration.

Figure 2a,b illustrates the registration, heartbeat, and inference workflows. Inference servers register their resources and periodically send heartbeats to update the orchestration server’s network view. Clients query the available models and submit inference requests through MCP. The orchestration server forwards each request to the HTTP–Petals inference backend and returns the generated output to the client.

### 3.2. Dynamic Model Selection

Let N(t) denote the network state at time *t*, consisting of the set of registered inference servers, their available GPU memory, compute capacity, downloaded model segments, and inter-peer latency estimates as reported during calls to SERVER_HEARTBEAT. We consider a set of candidate models $M=\{m_{1},m_{2},\ldots ,m_{K}\}$, ordered according to their GPU-memory requirements as $G_{1}\leq G_{2}\leq \cdots \leq G_{K}$, where $G_{j}$ denotes the memory required to host model $m_{j}$ under the same numerical precision.

Let $G_{\mathrm{edge}}$ denote the aggregate GPU memory that the stable edge servers can dedicate to collaborative inference. We assume that at least the smallest candidate model can be supported by the stable edge servers, i.e., $G_{1}\leq G_{\mathrm{edge}}$. We define the model-size boundary as

(1) $$i^{*}=\max\{i|G_{i}\leq G_{\mathrm{edge}}\}.$$

Accordingly, models $m_{1},\ldots ,m_{i^{*}}$ are referred to as smaller models, since they can be fully supported by the stable edge infrastructure without relying on mobile peers. Models $m_{i^{*}+1},\ldots ,m_{K}$ are referred to as larger models, since their memory requirements exceed the stable edge capacity and therefore require additional GPU resources contributed by nearby mobile robots.

Then, to address the second challenge, we introduce a procedure T(N) that determines, based on the evolution of the network state, when model redistribution should occur. Using this procedure, the MCP server can schedule model redistribution to minimize disruption to active inference requests and maximize utilization of available resources. Note that this is distinct from the problem of replacing peers that leave the network. As a simple example, T may be implemented as a time-of-day schedule, where the MCP server anticipates changes in available compute based on historical peer availability. At designated redistribution times, the MCP server must determine how to allocate existing computational resources. We define a memory utilization threshold γ, representing the maximum proportion of available GPU memory across peers that should be actively allocated to model segments. By keeping utilization below γ, the system reserves spare GPU memory for temporary changes in resource availability and for the predictive backup mechanism introduced in Section 3.3. Additionally, we define a policy π(N,γ) that determines the set of models that should be loaded within the γ proportion of available memory. Together, T, γ, and π provide the MCP server with a framework for resource management over time. The remaining question is how to optimally partition the selected models amongst the available computing resources. This may be addressed by adapting solutions such as that proposed by Mohammed et al. [26], which we leave for future work.

### 3.3. Predictive Backup for Model Partitions

As defined in Section 3.2, the smaller models are fully supported by stable edge servers and do not rely on mobile peers. Therefore, the predictive backup mechanism is needed only for larger models that distribute part of their model partitions to mobile robots. Immediately reconfiguring the entire model after every peer departure can introduce substantial delay. We therefore use the memory reserved by 1−γ to preload selected model partitions on backup workers. In contrast to uniform replication, which duplicates every partition hosted by a mobile worker, our policy prioritizes workers that are expected to leave the current orchestration region soon.

For a mobile worker *i*, let $\delta_{i}\left(t\right)$ denote the remaining path distance before the worker exits the current orchestration region, and let $v_{i}^{\mathrm{out}}\left(t\right)$ denote its planned outward velocity. We estimate its remaining residence time as

(2) $${\hat{T}}_{i}^{\mathrm{stay}}\left(t\right)=\left\{\begin{array}{cc} \frac{\delta_{i}\left(t\right)}{\max\;\left(v_{i}^{\mathrm{out}}\left(t\right),\epsilon \right)},\; & v_{i}^{\mathrm{out}}\left(t\right)>0,\\ +\infty ,\; & \mathrm{otherwise}, \end{array}\right.$$

where ϵ>0 avoids division by zero. Fixed edge servers are assigned ${\hat{T}}_{i}^{\mathrm{stay}}\left(t\right)=+\infty$. When a planned trajectory is unavailable, $\delta_{i}\left(t\right)$ can be conservatively estimated using the distance to a calibrated network boundary and the maximum robot speed.

Mobile workers are ranked according to their estimated remaining residence time ${\hat{T}}_{i}^{\mathrm{stay}}$, from the shortest to the longest. A shorter residence time indicates that the worker is more likely to leave the current orchestration region in the near future.

A backup is placed on a different worker with sufficient reserved memory. The server first prefers a fixed edge worker. Otherwise, it selects the mobile worker with the largest estimated residence time. If the reserved memory cannot accommodate every requested backup, partitions are processed in ascending order of the primary worker’s estimated residence time, so that the most imminent departures are protected first. When a mobile robot becomes unavailable, the orchestration server replaces its active partitions with the corresponding preloaded backups. If a complete backup path cannot be constructed, the server triggers the model adaptation procedure.

Figure 2c illustrates the failover path. When a primary worker is expected to leave, its partition is preloaded on a backup worker. After the departure is detected, subsequent inference requests use the preloaded backup partition. The remaining model partitions continue to run on their assigned workers.

## 4. A Running Example

Consider an autonomous vehicle performing a road inspection task in an urban area. During driving, the vehicle’s perception module converts the current sensor and task state into a textual prompt. The vehicle submits this prompt to an LLM. The LLM can use these data to interpret the environment and determine its next action. However, its onboard GPU memory is insufficient to execute the selected model locally. The vehicle therefore acts as an inference client and connects to a nearby MCP orchestration server.

The vehicle first uses QUERY_RTT to compare the latency of nearby orchestration servers and selects the one with the lowest round-trip time. It then uses QUERY_MODELS to obtain the currently available models and submits its inference request through PERFORM_INFERENCE.

The selected orchestration server maintains information about nearby inference servers, including edge servers and GPU-equipped vehicles. For example, two edge servers and parked or charging vehicles may register their available GPU memory, computing capacity, and downloaded model partitions through the REGISTER_SERVER interface. Based on this information, the orchestration server selects an appropriate model and assigns different transformer blocks to the available inference servers. During inference, the intermediate activations are forwarded through these servers in sequence. The final model output is returned to the requesting vehicle through the orchestration server.

The available computing resources may vary over time. Late at night or early in the morning, only a small number of vehicles may be available as inference servers, so a smaller model is maintained on stable edge servers. During the daytime, more GPU-equipped vehicles may register with the orchestration server. The server may then deploy a larger model across both edge servers and mobile peers. To tolerate anticipated peer departures, selected partitions hosted by mobile peers may be preloaded on backup workers, prioritizing peers with shorter estimated remaining residence times. If a protected peer leaves the network, the corresponding backup worker can serve its assigned partitions without immediately redistributing the entire model. The orchestration server detects resource changes through registration, deregistration, and heartbeat messages. Based on the model-redistribution procedure T, it determines when the deployed model set should be updated. The memory utilization threshold γ reserves sufficient capacity for redundancy. If the available resources are not enough for the larger model and its redundant partitions, the server switches back to the smaller model hosted by the fixed edge servers.

## 5. Implementation

We implemented the proposed architecture as three modules: an MCP client running on the robotic platform, a centralized MCP server, and a collaborative inference backend.

The control interface between the robotic systems and the backend is implemented using the MCP. The MCP client runs on the robotic platform and issues inference requests as PERFORM_INFERENCE tool calls. The centralized MCP server serves as the single entry point for all requests.

The distributed inference backend is implemented using Petals [24], an open-source framework that enables collaborative execution of large transformer models across multiple independent GPU servers. The source code of Petals is publicly available at https://github.com/bigscience-workshop/petals (accessed on 1 November 2026). However, its source code has not been maintained in the past two years and cannot be compiled correctly with the recent NVIDIA CUDA version. We have identified and fixed the bugs for our prototype. In Petals, model layers are hosted on remote workers and executed collectively through a distributed hash table (DHT). Workers that share the same *DHT prefix* form an isolated collaborative cluster. The optional peer configuration specifies bootstrap nodes for joining the distributed network.

The centralized MCP server also acts as a Petals client. Upon receiving an inference request, it selects the requested model configuration and forwards the generation request to the Petals backend. The server itself does not host the model weights. Instead, distributed execution is carried out by remote workers running Petals’ server API. All latency metrics reported in Section 6 are measured end-to-end at the robotic-side MCP client and include MCP communication overhead.

## 6. Evaluation

### 6.1. Experimental Setup

We evaluate collaborative inference across three model sizes and heterogeneous configurations of the available devices. Our computational resources comprise two 12 GB NVIDIA 4070 Ti GPU servers acting as edge-side Petals servers, two 8 GB Jetson Orin Nano boards, and one 64 GB Jetson AGX Orin board representing robot-side computational units. The GPU servers provide high-bandwidth, high-capacity inference capability, while the Jetson devices simulate realistic robotic computing constraints with limited memory and processing power. All devices are connected via a local WiFi network in a laboratory environment, representing typical edge network conditions where devices communicate over wireless links. Given the constraints of our current facilities, we only evaluated “heterogeneity” using computing devices equipped with different Nvidia GPUs, different memory capacities, and computational capabilities. We measure the round-trip time (RTT) between the devices. The mean RTT is 3.952 ms over 50 ping probes. The mean TCP throughput between the devices is 105.14 Mbps over three 10-s tests.

We evaluate three model configurations: **Llama-3.2-3B-Instruct**, **Meta-Llama-3-8B-Instruct**, and **Llama-2-13B**, using FP16 model weights. The 3B model represents a resource-constrained deployment scenario in which the entire model can be loaded on available devices even with limited edge participation. The 8B and 13B models simulate capacity-demanding scenarios that require collaborative inference between multiple devices. These experiments characterize collaborative inference across different model sizes and device configurations.

### 6.2. Performance of Different Configurations

We generate 10 prompts that cover various mobile robotics scenarios. Then, for each topology configuration, we test each of the 10 prompts 5 times, yielding 50 inference requests per configuration to ensure statistical reliability. Each request specifies a maximum generation length of 64 tokens with a timeout of 180 s. Before measured requests are executed, we execute a single warm-up request to ensure model loading and network establishment are excluded from performance measurements. This warm-start methodology isolates steady-state inference performance from cold-start overheads. The additional 13B evaluation follows the same prompt, repetition, generation-limit, timeout, and warm-up protocol. We evaluate four collaborative configurations, using identical block boundaries for configurations with the same number of participating devices.

Table 1 presents the performance evaluation in 14 topology configurations. The block partition column indicates how transformer layers are distributed across devices, with ranges like 0–9, 10–22, 23–27 showing consecutive layer assignments to different nodes in the collaborative network. The block intervals follow the device order in the topology label: T1 and T2 first, followed by the listed Jetson devices. For example, in 2T+1J, the first two intervals are hosted by T1 and T2, respectively, and the final interval by J. We calculate the average value out of 50 requests. For Time-to-First-Token (TTFT) and total response time, each cell reports the mean, sample standard deviation, and P95 over 50 measured requests. Except for the 1AGX robot-only baseline, all collaborative configurations include at least one edge server.

These results demonstrate different performance characteristics across different inference server configurations. When a robot operates independently using only an AGX processor, inference achieves 1.034 s TTFT for the 3B model. Adding collaboration with both edge servers (i.e., the 2T+1AGX configuration) improves this to 0.640 s, demonstrating the benefit of offloading computation to shared edge infrastructure. For the 8B model, the baseline robot-only 1AGX configuration has a TTFT of 0.956 s. The 2T+1AGX collaborative configuration achieves 0.565 s, showing substantial improvement when edge servers assist the robot’s GPU.

Robots equipped with Jetson Nano processors have different performance characteristics. With the 3B model, the 2T+1J configuration achieves 0.650 s TTFT, similar to the AGX-based 2T+1AGX at 0.640 s. However, for the larger 8B model, the Jetson Nano configuration degrades to 0.645 s compared to the AGX at 0.565 s. This difference reflects the Jetson Nano’s lower memory bandwidth and reduced TFLOPS, which create constraints when handling larger model partitions in collaborative inference.

Across both model sizes, the topology design and network characteristics influence performance significantly more than model size alone. Device composition (e.g., including high-performance GPUs and capable edge processors), block placement, and communication overhead can all affect TTFT. On the 2T+1AGX topology, the 8B model achieves 0.565 s TTFT compared to the 3B model’s 0.640 s. However, examining token generation rate reveals a more nuanced picture: the 8B model achieves 6.43 tok/s versus the 3B model’s 6.98 tok/s on the same topology—a natural consequence of increased computation. This observation indicates that TTFT does not increase monotonically with model size under the tested deployment configurations. In contrast, the 3B model on the 2T+2J topology (with less capable Jetson Nano processors) achieves 1.110 s TTFT and 4.48 tok/s, demonstrating that processor capability and topology design significantly impact both latency and throughput. This shows that the collaborative framework enables using larger, more capable models without excessive latency penalty: despite 2.67× more parameters, the 8B model’s throughput (6.43 tok/s) remains practical. The Llama-2-13B evaluation extends this comparison to a larger model. Across the four collaborative topologies, its mean TTFT ranges from 1.080 to 3.724 s, with output speeds ranging from 2.95 to 5.67 tok/s. The 13B model also generates longer responses (54.5 tokens on average, compared with approximately 40 tokens for 8B), which contributes to its longer total response time.

### 6.3. MCP Invocation Overhead

As the MCP implementation [27] we used is built on top of HTTP, we measure the additional overhead brought by MCP by comparing direct HTTP requests with MCP tool calls to the same Petals inference service. Both tests run Llama-3.2-3B-Instruct in FP16 on two 4070 Ti servers and one 8 GB Jetson Orin Nano board, hosting blocks 0–9, 10–23, and 24–27, respectively. After one warm-up request per path, we execute 30 request pairs using the same prompts and a maximum output length of 64 tokens. Each pair uses the same prompt for both paths. For each request, we record both the total response time and the inference time. We subtract the processing time from the total response time in each test, and estimate MCP overhead as the additional time. Each MCP call opens a new session, so this estimate includes connection and session setup. The measured MCP overhead averages 39.71 ms, with a sample standard deviation of 3.08 ms and a P95 of 44.72 ms across the 30 request pairs.

### 6.4. Dynamic Model Switching

Next, we evaluate the overall system overhead during online model switching. The model upgrade scenario represents a case in which additional GPU memory is available within the active γ proportion of the total GPU memory, allowing model redistribution to switch the system to a larger model. In this scenario, the system initially runs the 3B model on three nodes: A, B (both 4070 Ti edge servers) and C (Jetson AGX vehicle). After the first 10 requests, the redistribution is triggered, and the 8B model runs across nodes A, B, C, and D, where D is a Jetson Nano powered robot. On the other hand, the model fallback scenario represents a case where less GPU memory is available during redistribution. In this case, the system initially runs the 8B model on all four nodes (A, B, C, and D), and after the first 10 requests, the system reconfigures back to the 3B model running on nodes A, B, and C.

Each scenario consists of 30 measured inference requests. The model switch is triggered at request 11. Requests 1–10 run under the initial model configuration to establish steady-state baseline performance, request 11 executes the cluster reconfiguration and runs under the new model, and requests 12–30 continue under the new model configuration. In the performance analysis, “3B steady” or “8B steady” refers to the average performance over the first 10 requests before switching, “Switch” refers to the single switching request (request 11) that bears the reconfiguration overhead, and the final steady state (“8B steady” for upgrade or “3B steady” for fallback) represents the average performance over requests 12–30 after the system stabilizes under the new configuration. This evaluation simulates continuously arriving requests across model transitions to measure the system’s dynamic adaptation capability.

The phase-wise results in Table 2 show three phases for each scenario: the initial steady state before switching, the switching phase during model reconfiguration, and the final steady state after switching. In the upgrade scenario, the system begins with the 3B model running at 0.623 s TTFT on three nodes. During the model switch to the 8B model, performance temporarily degrades to 2.761 s TTFT as layers are redistributed and reloaded. Once the system stabilizes on the 8B model with four nodes, the TTFT settles to 1.159 s. This result demonstrates that despite handling a larger model, the additional compute node enables practical performance after switching overhead subsides. The token generation rates reflect this pattern: 7.00 tok/s initially, dropping to 2.10 tok/s during the switch, and recovering to 3.76 tok/s in the final 8B configuration. The fallback scenario shows the inverse pattern. The system runs the 8B model at 1.052 s TTFT on four nodes. When a node leaves and the system switches to the 3B model on three nodes, performance improves substantially: TTFT decreases to 0.611 s in the final state, with token generation improving from 3.68 to 7.31 tok/s. The temporary switching phase shows an intermediate latency of 1.497 s as node D’s responsibility is offloaded.

Table 3 breaks down the switching overhead into components. The **Cmd** column measures the cluster reconfiguration command execution time: 3.58 s for the upgrade case and 3.47 s for the fallback case. The **1st Token** column represents the elapsed time from the start of the switch command until the first output token is generated under the new model configuration, calculated as the command execution time plus the TTFT of the first inference request under the new configuration. Similarly, the **Response** column represents the elapsed time from the start of the switch command until the complete response is returned, calculated as the command execution time plus the inference latency of the switching request. In both scenarios, all 30 inference requests are completed successfully, demonstrating that the system handles model transitions without failures. The upgrade case incurs higher switching latency, with 6.34 s to the first token and 27.17 s to the complete response, compared with 4.97 and 15.07 s, respectively, in the fallback case. This is expected given the additional complexity of adding a new node and loading a larger model.

We additionally evaluate repeated model switching using the sequence 3B → 8B → 3B → 8B, with 20 inference requests per stage and a maximum output length of 64 tokens. The 3B model runs on the two edge servers, whereas the 8B model additionally uses the 8 GB Jetson Orin Nano. All three transitions complete, and all 80 measured requests succeed. Table 4 separates the first request after each switch from subsequent steady-state requests. The device configurations differ from those in the preceding switching experiment, so the latency values are not directly comparable. Total denotes the complete MCP request time. Steady-state values are averages over 20 requests in the initial stage and 19 requests in each subsequent stage. Each first request row represents one request. After each switch, request latency decreases from the first-request level to similar steady-state values for the same model, demonstrating repeated model switching and subsequent inference.

### 6.5. Evaluation of Dynamic Model Selection

The preceding hardware experiment triggers model switching at a predefined request. We additionally evaluate automatic model selection under repeated changes in available resources, using a concrete implementation of T(N) and π(N,γ) from Section 3.2. The purpose is to test whether changes in the network state can trigger model redistribution without manually specifying when to switch.

We simulate the controlled worker joining/leaving using two 4070 Ti edge servers (T1 and T2) and one Jetson Orin Nano with 8 GB of shared memory. These events update N(t), including the registered servers, their contributed memory budgets, and cached models. Heartbeats are sent every 3 s, and a server is removed from the registered set after 12 s without a heartbeat. Explicit deregistration removes it directly. Registration and heartbeat messages are collected through persistent network connections, while inference uses the MCP–HTTP–Petals pipeline. The candidate set M contains the 3B and 8B models. Each fixed edge server contributes a service memory budget of 8589 MB, and the Jetson contributes 6442 MB. Under these settings, the two fixed edge servers support the 3B configuration, whereas the 8B configuration requires the additional Jetson worker.

Given N(t), π(N,γ) selects the largest candidate with a memory-feasible block assignment, using γ=0.8. We use two validated contiguous placement configurations: for 3B, T1 and T2 host blocks 0–13 and 14–27, respectively; for 8B, T1, T2, and the Jetson host blocks 0–11, 12–23, and 24–31, respectively. Feasibility checks include the FP16 block weights and a 1610 MB runtime allowance per worker. The procedure T(N) triggers an upgrade only after the resources required by 8B have remained available for 5 s, subject to a 15 s cooldown after a committed deployment. If a departure makes the active placement infeasible, redistribution bypasses these upgrade delays and selects the feasible 3B configuration. A deployment is committed only after all assigned blocks are reported online on the designated workers.

We analyze the first 15 upgrade–fallback tests, including 15 transitions from 3B to 8B and 15 transitions from 8B to 3B. All 30 transitions complete. After each transition, an inference request with a maximum generation length of 64 tokens and a timeout of 180 s verifies execution using the assigned workers. All 30 post-transition requests succeed. These results demonstrate the feasibility of dynamic model selection and deployment on the physical testbed.

### 6.6. Predictive Partition Backup

#### 6.6.1. Comparison with and Without Predictive Partition Backup

We evaluate predictive partition backup using FP16 Llama-3.2-3B-Instruct on the 2T+2J configuration. Servers A and B host blocks 0–9 and 10–17, while Jetsons C and D host blocks 18–22 and 23–27. We simulate D leaving the network by stopping it after request 10. In the test, the backup policy uses the estimated remaining residence times ${\hat{T}}_{i}^{\mathrm{stay}}$ to select D’s partition for backup on C. We compare two cases: with and without predictive partition backup. With predictive partition backup, C loads D’s blocks before departure and takes over afterward. Without predictive partition backup, C starts loading these blocks when the first post-departure request arrives. Both tests execute partition loading and inference on real hardware.

Each test uses at most 64 output tokens per request. Table 5 reports the recovery waiting time and complete response time of the first request after the controlled departure. Complete response time is measured from the MCP client invocation which includes recovery waiting time, inference time, and communication overhead. Predictive partition backup reduces the observed recovery waiting time from 19.802 to 0.003 s. The complete response time decreases from 34.752 to 11.496 s. By preparing D’s partition on C before departure, the mechanism avoids loading the partition on demand and enables faster takeover.

#### 6.6.2. Repeated Worker Departures and Rejoins

We further test predictive partition backup over five consecutive departure–rejoin tests using the same 3B model and block assignments. In each test, D serves the first two requests, C’s preloaded backup serves five requests after D leaves, and D serves three requests after returning. D rejoins inference after its assigned blocks are ready. All the tests complete, including five departures and five rejoins, and all 50 requests succeed.

Table 6 summarizes recovery waiting and complete response times across the five tests, using the same timing definitions as the preceding comparison. The first requests after departure and rejoin generate 37 and 64 tokens, respectively. These results demonstrate repeated backup takeover and worker rejoining with predictive partition backup.

## 7. Related Work

### 7.1. Distributed LLM Inference and Serving

Recent LLM serving systems show that inference performance is shaped by prompt processing and autoregressive decoding. DistServe [28] separates prefill and decoding to improve goodput and SLO attainment. ServerlessLLM [29] addresses low-latency LLM inference under serverless constraints via fast loading and migration. vLLM [30] improves throughput by managing the KV cache with OS-inspired paging. Several systems focus on multiplexing and heterogeneity. MuxServe [31] multiplexes multiple LLMs and exploits prefill and decoding structure for placement and scheduling. Helix [32] optimizes placement and scheduling in heterogeneous GPU clusters using a max-flow formulation. Aegaeon [33] studies GPU pooling and introduces token-level scheduling and autoscaling for concurrent LLM serving.

### 7.2. Collaborative LLM Inference on the Edge and Mobile Devices

Edge deployment adds strict memory limits and device heterogeneity. Jupiter [34] targets collaborative generative LLM inference on edge devices and explicitly optimizes both prefill and decoding. EdgeShard [35] formulates joint device selection and model partitioning for collaborative LLM inference across heterogeneous nodes. LinguaLinked [36] distributes LLM segments across mobile devices and addresses runtime load balancing. HPipe [37] proposes pipeline parallelism on heterogeneous commodity devices for long-context inference. Mobility-aware work such as MoEI [38] highlights that migration and topology change can dominate latency. In contrast to these works, we introduce the MCP orchestration servers, which create a set of stable points in the network topology that enable a transparent interface for both clients and inference servers.

### 7.3. LLM and VLA Models in Robotics

Robotic pipelines are increasingly relying on large foundation models. Code as Policies generates executable robot policy code from language [20]. Large embodied multimodal models such as PaLM-E [13] reach 562B parameters, increasing deployment pressure for onboard robots and motivating system support beyond on-device inference. Vision-language-action models such as RT-2 [21] and generalist transformers such as OpenVLA [22] demonstrate strong generalization but increase the demand for efficient inference infrastructure. Planning-focused systems such as SELP [39] show that robotic task planning often requires explicit constraint representations and constrained decoding to enforce safety. To address the need for inference on larger models than can be used locally, our work proposes integrating collaborative inference between the robotic systems and edge servers, decreasing reliance on high latency cloud services and improving the utilization of the nearby computing resources.

## 8. Discussion

### 8.1. Trust Assumptions and Open-Environment Deployment

The current prototype considers collaborative inference among trusted devices, such as robots and edge servers managed by the same organization. This deployment setting allows us to investigate system orchestration and inference performance without introducing an incentive mechanism for independent participants. Extending the framework to open environments would require additional mechanisms to address malicious peers, the privacy of intermediate activations, and the integrity of inference results. Intermediate activations may reveal sensitive input information. A standardized MCP interface does not by itself establish the trustworthiness of participating devices or their outputs. These considerations motivate future work on secure collaboration among untrusted participants.

### 8.2. Testbed Representativeness

The testbed represents an edge-assisted multi-robot deployment in which fixed edge servers share computing resources with robot-side computers. The edge servers represent the fixed infrastructure, while the Jetson devices represent robots with different memory capacities and computational capabilities. We simulate robots leaving and returning to the network by stopping and restarting their inference workers. Model reconfiguration, partition loading, backup takeover, and inference are executed on real hardware. This setup allows us to evaluate how collaborative inference responds to the loss and return of robot-side computing resources, while physical movement and the resulting wireless-channel changes are not reproduced.

### 8.3. Application Scenarios and Latency Considerations

The framework targets high-level decisions in mobile robotic applications, such as selecting the next task or generating a subsequent task plan in an industrial environment. Inference can be requested in advance while a robot executes its current task or travels along an established route. This allows inference latency to overlap with ongoing task execution and can reduce additional waiting time when the result is available before the next decision is needed. In such a deployment, a local controller would continue executing the current valid plan while inference or model reconfiguration is in progress. New plans would be applied only after a response is available and checked by the application. These are possible application-level deployment strategies.

## 9. Conclusions

In this work, we have proposed a framework for collaborative LLM inference in mobile robot deployments that combines an MCP-based orchestration interface with dynamic model selection and predictive backup for model partitions. Our framework has been carefully designed to accommodate mobile robot deployments and we demonstrate the feasibility of collaborative inference under a controlled laboratory setting. The experimental evaluation demonstrates the efficiency of our proposed framework.

## Figures and Tables

**Table 1:** End-to-end performance summary across 3B/8B/13B models and different topologies. T denotes the 4070 Ti GPU servers, J represents Jetson Nano boards, and AGX refers to the Jetson AGX Orin board. TTFT and total time are reported as mean ± sample standard deviation (P95) over 50 requests per configuration.

Model	Topology	Block Partition	TTFT (s)	Output Speed (Tok/s)	Gen. Tokens	Total Time (s)
Llama-3.2-3B-Instruct	1AGX	0–27	1.034±0.084(1.187)	6.32	36.1	6.05±2.48(6.84)
Llama-3.2-3B-Instruct	2T+1AGX	0–9, 10–22, 23–27	0.640±0.090(0.668)	6.98	36.3	5.33±0.85(6.41)
Llama-3.2-3B-Instruct	2T+1J	0–9, 10–22, 23–27	0.650±0.040(0.723)	6.94	36.3	5.40±1.13(6.54)
Llama-3.2-3B-Instruct	2T+1J+1AGX	0–9, 10–17, 18–22, 23–27	1.235±0.121(1.486)	4.31	36.7	8.76±1.56(11.30)
Llama-3.2-3B-Instruct	2T+2J	0–9, 10–17, 18–22, 23–27	1.110±0.104(1.316)	4.48	36.5	8.37±1.84(9.92)
Meta-Llama-3-8B-Instruct	1AGX	0–31	0.956±0.146(1.170)	4.47	39.7	9.24±3.04(10.87)
Meta-Llama-3-8B-Instruct	2T+1AGX	0–9, 10–27, 28–31	0.565±0.051(0.652)	6.43	40.0	6.32±0.77(7.83)
Meta-Llama-3-8B-Instruct	2T+1J	0–9, 10–27, 28–31	0.645±0.125(0.802)	6.22	40.0	6.61±1.37(7.52)
Meta-Llama-3-8B-Instruct	2T+1J+1AGX	0–9, 10–23, 24–27, 28–31	1.045±0.109(1.303)	3.91	39.8	10.36±1.59(12.21)
Meta-Llama-3-8B-Instruct	2T+2J	0–9, 10–23, 24–27, 28–31	1.150±0.209(1.559)	3.76	39.8	10.80±1.83(12.81)
Llama-2-13B	2T+1AGX	0–15, 16–33, 34–39	1.080±0.083(1.207)	5.67	54.5	9.676±0.867(11.062)
Llama-2-13B	2T+1J	0–15, 16–33, 34–39	1.828±0.268(2.245)	4.55	54.5	12.028±0.807(13.158)
Llama-2-13B	2T+1J+1AGX	0–12, 13–29, 30–34, 35–39	2.446±0.286(2.926)	3.03	54.5	18.029±1.382(19.712)
Llama-2-13B	2T+2J	0–12, 13–29, 30–34, 35–39	3.724±0.336(4.305)	2.95	54.5	18.529±1.403(20.826)

**Table 2:** Performance during steady-state and model switching phases.

Case	Phase	TTFT (s)	Total (s)	Tok/s
Model upgrade	3B steady	0.623	5.527	7.00
Model upgrade	Switch	2.761	23.589	2.10
Model upgrade	8B steady	1.159	10.676	3.76
Model fallback	8B steady	1.052	11.241	3.68
Model fallback	Switch	1.497	11.600	3.51
Model fallback	3B steady	0.611	5.080	7.31

**Table 3:** Dynamic model-switching overhead results.

Case	Cmd (s)	1st Token (s)	Response (s)	Success
Model upgrade	3.58	6.34	27.17	30/30
Model fallback	3.47	4.97	15.07	30/30

**Table 4:** Request performance during repeated model switching.

Stage	Phase	TTFT (s)	Total (s)	Tok/s
Initial 3B	Steady	0.288	1.630	22.74
3B → 8B	First request	2.129	17.617	2.66
	8B steady	0.961	6.957	5.75
8B → 3B	First request	0.698	5.947	7.61
	3B steady	0.289	1.662	22.25
3B → 8B	First request	2.371	17.767	2.60
	8B steady	0.940	7.024	5.71

**Table 5:** Time cost of first post-departure request with and without predictive partition backup.

Metric	Without Predictive Partition Backup	With Predictive Partition Backup
Recovery waiting time (s)	19.802	0.003
Complete response time (s)	34.752	11.496

**Table 6:** Predictive partition backup over five departure–rejoin tests. Values are mean ± sample SD (P95) across five tests.

Metric	With Predictive Partition Backup
Recovery waiting time after departure (s)	0.00250±0.00019(0.00269)
Complete response time after departure (s)	9.185±1.605(11.141)
Complete response time after rejoin (s)	14.314±0.937(15.506)

## Data Availability

The original contributions presented in this study are included in the article. Further inquiries can be directed to the corresponding author.
